# Roles of Arbuscular Mycorrhizal Fungi on Soil Fertility: Contribution in the Improvement of Physical, Chemical, and Biological Properties of the Soil

**DOI:** 10.3389/ffunb.2022.723892

**Published:** 2022-03-07

**Authors:** Abdoulaye Fofana Fall, Grace Nakabonge, Joseph Ssekandi, Hassna Founoune-Mboup, Samuel Obeng Apori, Abibatou Ndiaye, Arfang Badji, Khady Ngom

**Affiliations:** ^1^African Center of Excellence in Agroecology and Livelihood Systems, Faculty of Agriculture, Uganda Martyrs University, Nkozi, Uganda; ^2^Fungi Biotechnology Laboratory, Plant Biology Department, Cheikh Anta Diop University of Dakar (UCAD), Dakar, Senegal; ^3^College of Agriculture and Environmental Sciences, Makerere University, Kampala, Uganda; ^4^ISRA_LNRPV, Laboratoire National de Recherches sur les Productions Végétales (LNRPV), Dakar, Senegal; ^5^School of Food Science and Environmental Health, Technological University Dublin, Dublin, Ireland; ^6^Department of Agricultural Production, Makerere University, Kampala, Uganda

**Keywords:** macro-aggregation, microorganisms, glomalin, rock phosphate, P legacy

## Abstract

Many of the world's soils are experiencing degradation at an alarming rate. Climate change and some agricultural management practices, such as tillage and excessive use of chemicals, have all contributed to the degradation of soil fertility. Arbuscular Mycorrhizal Fungi (AMFs) contribute to the improvement of soil fertility. Here, a short review focusing on the role of AMF in improving soil fertility is presented. The aim of this review was to explore the role of AMF in improving the chemical, physical, and biological properties of the soil. We highlight some beneficial effects of AMF on soil carbon sequestration, nutrient contents, microbial activities, and soil structure. AMF has a positive impact on the soil by producing organic acids and glomalin, which protect from soil erosion, chelate heavy metals, improve carbon sequestration, and stabilize soil macro-aggregation. AMF also recruits bacteria that produce alkaline phosphatase, a mineralization soil enzyme associated with organic phosphorus availability. Moreover, AMFs influence the composition, diversity, and activity of microbial communities in the soil through mechanisms of antagonism or cooperation. All of these AMF activities contribute to improve soil fertility. Knowledge gaps are identified and discussed in the context of future research in this review. This will help us better understand AMF, stimulate further research, and help in sustaining the soil fertility.

## Introduction

**Figure 1:**
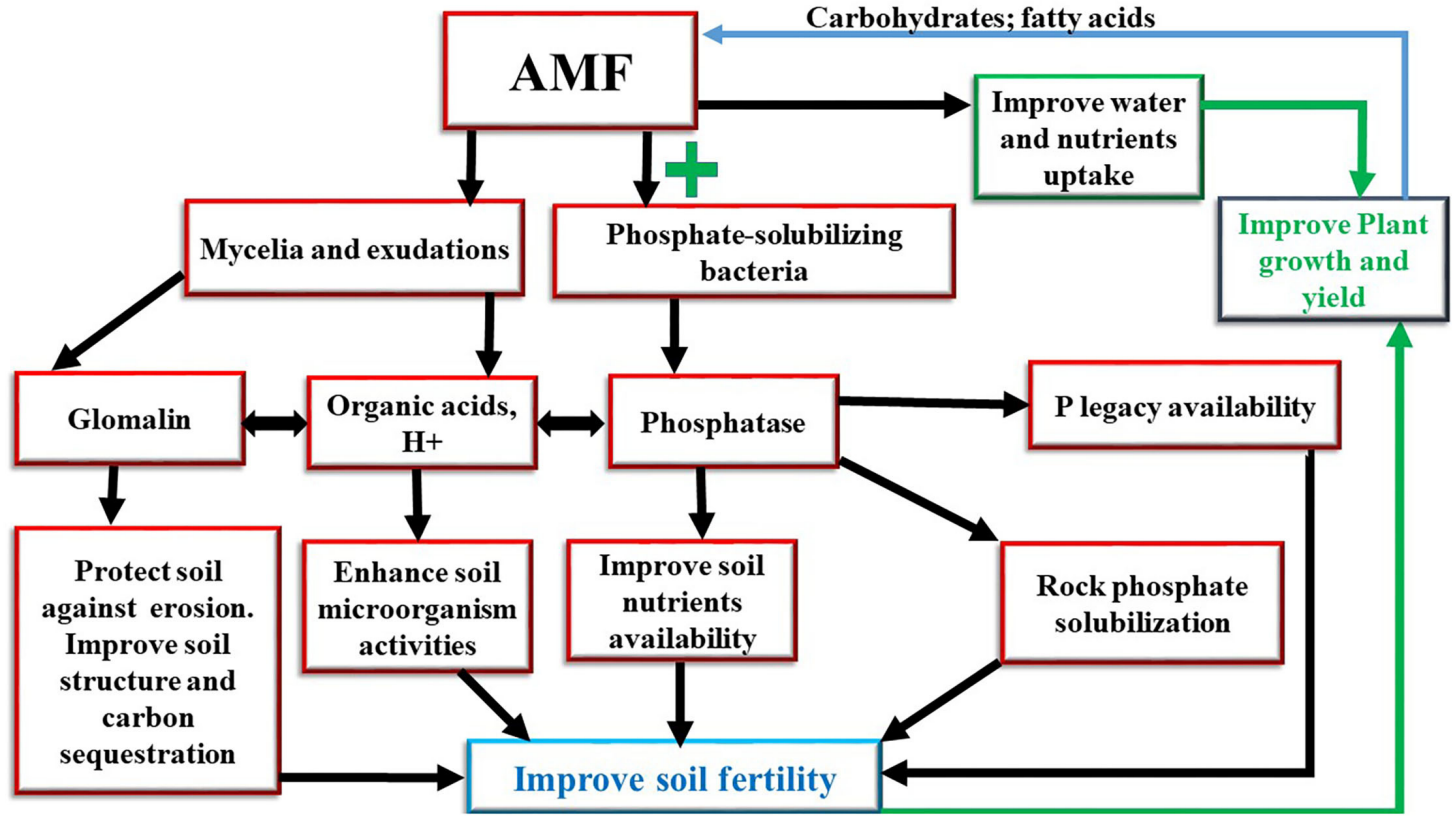
Effects of arbuscular mycorrhizal fungi on improving soil fertility.

The most significant threats to soil function at the global level are soil erosion, soil organic carbon, excessive use of input, and nutrient imbalance (Montanarella et al., 2016). The depletion of soil fertility in the world has increased due to unsustainable land management practices, such as overgrazing, bush burning, continuous crop cultivations, and tillage practices (Dewitte et al., 2013). However, inoculation with Arbuscular Mycorrhizae Fungi (AMFs) has been identified as an eco-friendly approach to improve soil fertility (Dal Cortivo et al., 2018). AMF is the most widespread soil microorganisms that form a symbiotic relationship with more than 80% of plants (Prasad et al., 2017), except for a few plant families, such as *Amaranthaceae, Brassicaceae, Cruciferae, Chenopodiaceae, Caryophyllaceae, Juncaceae, Cyperaceae*, and *Polygonaceae*, which do not exhibit any association (Brundrett, 2009). They can be found in various ecosystems worldwide (Verbruggen et al., 2012). AMF is a key component of soil microorganisms and belongs to the *glomeromycota* phylum. This phylum is divided into three classes (Archaeosporomycetes, Glomeromycetes, and Paraglomeromycetes), five orders (*Archaeosporales, Diversisporales, Gigasporales, Glomerales*, and *Paraglomerales*), 14 families, 29 genera, and more than 240 species (Krüger et al., 2012; Redecker et al., 2013). Several species of AMF have been studied in the world, however, the most species used as a model are as follows: *Funneliformis mosseae* (previously known as *Glomus mosseae*), *Gigaspora rosea, Gigaspora margarita, Gigaspora gigantea*, and *Rhizophagus irregularis* (previously known as *Glomus intraradices* and *Glomus irregulare*; Schüßler and Walker, 2010). AMF is not a parasite but obligate symbionts that need a host plant to complete their life cycle. They improve crop productivity by increasing water and nutrient uptake, such as nitrogen (N), phosphorus (P), and potassium (K) (Anderson et al., 2018). The increase of the host plant nutrient uptake is due to the characteristics of AMF mycelium. These mycelia or hyphae absorb nutrients by osmotrophy and explore more surface area compared to non-mycorrhizal roots (Duponnois et al., 2011). In return, AMF benefits carbohydrates from the host plants (Diagne et al., 2020). Many authors demonstrated that AMF obtains up to 20% of photosynthetic carbohydrates from the host plant (Bonfante and Desirò, 2015; Kaiser et al., 2015). In addition to carbohydrates, lipids are a major source of organic carbon delivered to the fungus (Luginbuehl et al., 2017). It has been discovered that plants provide the fungus with some of the fatty acids that the microorganism needs to grow (Keymer et al., 2017). The biosynthesis of fatty acids has not been observed in AMF in the absence of the plant. Moreover, the genes encoding for fatty acid biosynthesis have not been found in AMF, therefore, these microorganisms depend on the lipid biosynthesis of the host plant. AMF is an extremely ancient symbiosis. Based on archeologic records, it dates to the appearance of terrestrial plants million years ago and would have accompanied vascular plants to colonize the terrestrial environment (Humphreys et al., 2010). AMF does not only have an impact on plant growth and production but it has been also reported that they improve some soil characteristics, such as soil aggregation, soil nutrients availability, water retention, microbial activities, nitrogen, carbon, and phosphorus cycling, and soil acidity correction (Sadhana, 2014; Jamiołkowska et al., 2018; Parihar et al., 2020). Several studies have reported that they play a crucial role in plant resistance against biotic and abiotic stresses. This review aims to summarize knowledge about AMF symbiosis, in particular, the beneficial effects on soil (Figure 1). First, the role of AMF in the physical, chemical, and biological properties of the soil is considered. The contribution of AMF in soil aggregation, nutrient availability, and boosting beneficial soil microorganisms is discussed. Finally, the role diversity of interactions between AMF and other soil microorganisms is examined.

## Role of AMF on Improving Soil Physical Properties

### Importance of AMF on Soil Structure

Arbuscular Mycorrhizae Fungi have a beneficial effect on soil structure. The AMF mycelia are present in massive quantities in soils (de Novais et al., 2019). These mycelia or hyphae have the property to create stable soil aggregations. Mycorrhizal fungi act as a long-term soil binding agent through the production of a glycoprotein (glomalin) by the extramatrical mycelia (Singh et al., 2020). This glomalin is a hydrophobic, thermo-tolerant, or heat-tolerant resistant to the hot temperature of the soil. The hydrophobic character of the glomalin confers resistance of soil aggregations to water, the production of this substance reaches its maximum in senescent mycelia. The glycoprotein is slowly biodegradable by bacteria and fungi in the soil. The main function of glomalin is to stabilize soil aggregations (Hu et al., 2019; Mubekaphi, 2019), act as a glue that binds together the soil micro-aggregations (diameter <250 μm) to form stable macro-aggregations (Lehmann et al., 2020). These soil macro-aggregations ensure better water infiltration, reduce surface runoff, control soil erosion, reduce nutrients and organic matter losses, increase gas exchange better retention of water and minerals, especially potassium, therefore, improve crop productivity (Demenois et al., 2018; Parihar et al., 2020). In addition, the mycelia network constantly renews itself and the dead mycelia also preserve soil structure until decomposition (Gianinazzi et al., 2010). These dead mycelia contribute to the stocks of organic matter and physical binder involved in soil aggregation (Hamel and Plenchette, 2017). All these mechanisms reduce the risks of soil compaction and promote soil fertility (Norton et al., 2020). It can be said that AMFs improve soil structure through their chemical and biophysical mechanisms, such as enmeshment and alignment. However, there is a lack of information about the lifespan of glomalin in the soil and the effect of anthropology activities, such as bush burning, on soil glomalin.

## Role of AMF on Improving Soil Chemical Properties

Arbuscular Mycorrhizae Fungi symbionts are recognized as being major microbial components in the development of the main biogeochemical cycles of soils (P, N, and C). This results in an improvement in the growth of mycorrhizal plants.

### Contribution of AMF on Soil Phosphorus Legacy Availability

Phosphorus is an essential element for plants. It is a component of many molecules, such as adenosine triphosphate (ATP), nucleotides, phospholipids, certain enzymes, and co-enzymes (Agledal et al., 2013). Most soils contain enormous amounts of organic and inorganic phosphorus (Requejo and Eichler-Löbermann, 2014). The accumulation of P in soils from fertilizers is known as legacy P (Sattari et al., 2012). This legacy P has the potential to play a key role in maintaining agricultural productivity (Condron et al., 2013; Rowe et al., 2016). It has been revealed that the accumulated P in soils is sufficient to sustain crop yields worldwide for about 100 years (Khan and Zaidi, 2007). Unfortunately, only a few quantities of this soil's P are available for the plants (Balemi and Negisho, 2012). The P is most often in the form of inorganic orthophosphate adsorbed to soil cations. Thus, the availability of P in soil is affected by the presence of iron (Fe), calcium (Ca), and aluminum (Al) oxides, which fix phosphorus as iron phosphate (FePO_4_), tri-calcium phosphate [Ca_3_(PO_4_)_2_], and aluminum phosphate (AlPO_4_) (Amanullah and Zakirullah, 2010; Shen et al., 2011). Therefore, only a small proportion (<1%) of the legacy P is available to plants (Rodrigues et al., 2021). Phosphorus is taken in the form of orthophosphates (inorganic phosphate Pi) by plants, but this mineral form of phosphorus is in limited quantity in the soil and, under the action of root sampling, areas are quickly created depletion around the roots due to a slow supply of P, slow phase of the soil, and the low mobility of P in soils (Javot et al., 2007). The reservoir of P must be hydrolyzed to make it available in the soil for plants uptake. AMF plays a key role in improving P availability in the soil. Indeed, it is a P activator that can accelerate the process to transform P into bio-available forms via a range of chemical reactions and biological interactions (Zhu et al., 2018). It was believed that AMF hydrolyzes the organic P into inorganic phosphorus (Shen et al., 2011) through a mechanism linked to the production of enzymes named phosphatase (Tarafdar and Marschner, 1994). However, recent studies revealed that AMF lack the capacity to release phosphatases into the soil (Zhang et al., 2016) but they recruit bacteria known as Phosphate Solubilizing Bacteria (PSB) that produce phosphatase, which mineralizes organic P and provides a function that is absent from the AMF (Zhang et al., 2018; Etesami and Jeong, 2021). PSB and AMF association is a beneficial feature that has the ability to mineralize insoluble phosphate in the soil and release soluble P that can easily be assimilated by plants (Wei et al., 2017; Mahanta et al., 2018). In that association, the role of PSB is to produce organic acids, such as gluconic acid, ketogluconic acids, siderophores, protons, and acid phosphatases that are involved in the mineralization of organic P in soil (Dobbelaere et al., 2003; Lucy et al., 2004), while AMF mycelia improve the absorption of soluble P in the plants (Taktek et al., 2017). The phosphatase releases P from organic P or inorganic orthophosphate by hydrolyzing phosphoric acid monoesters into P ion and a molecule with a free hydroxyl group (Othman and Panhwar, 2014). It was proven that the double inoculation of *R. irregularis* and *Rahnella aquatilis* improves solubilization of inorganic P by the increased production of phosphatase released by the bacteria that is also stimulated by AMF exuded fructose (Zhang et al., 2018). AMF can also solubilize inorganic phosphate into soluble forms through the processes of acidification, chelation, exchange reactions, and production of organic acids, H^+^, and metabolites (Relwani et al., 2008; Behera et al., 2014). It is demonstrated that the metabolic activities of AMF produce alkaline phosphatases, which cleave substrates present in the soil and make the phosphate accessible (Liu et al., 2013). Moreover, the organic acid produced by AMF solubilizes insoluble mineral phosphate into a soluble form (Lapeyrie, 1988). In addition, AMFs help to release P from rock phosphate (RP) fertilizer. RP has low effectiveness. This is due to when added as fertilizer only one part is accessible to the plants and the remaining part is converted into insoluble fixed forms (Billah et al., 2019). Thus, AMF can solubilize insoluble phosphate from RP to make it available in the soil (Andrino et al., 2021). AMF converts the insoluble P into soluble forms through their production of acids during their metabolic activities (Kalayu, 2019). However, little is known about whether there is an activator dose of P that allows AMF to initiate root infection. Because it is known that a rate of 50 kg N ha^−1^ is the starter dose to activate rhizobium symbiosis.

### Contribution of AMF on Soil Nitrogen Availability

Like phosphorus, nitrogen (N) is a vital part of plants. It is a constituent of phospholipids, coenzymes, and amino acids (Hawkesford et al., 2012). In the soil, N is present in organic and mineral forms (nitrites, nitrates, and ammonium ions). The ammonium form is weakly absorbed by plants that prefer nitrogen in the form of nitrate ($NO_{3}^{-}$). AMF helps to mobilize the inorganic form of nitrogen ($NO_{4}^{+}$ from the soil (Casieri et al., 2013). The AMF mycelium is able to absorb nitrogen in the form of ammonium ions ($NO_{4}^{+}$), in the form of nitrates ($NO_{3}^{-}$), and in the form of amino acids (Chen et al., 2018; Drechsler et al., 2018; Jansa et al., 2019). Nitrogen availability requires the activity of local transporters in the AMF hyphae. It has also been demonstrated that mycorrhizal associations could play a significant role in the decomposition and mineralization of plant organic matter and mobilize nutrients, particularly nitrogen, for the benefit of the host plant (Lambers et al., 2008). However, more research should be conducted in a controlled environment to determine the quantity of nitrogen that transits through the AMF mycelia network. Moreover, a study should be carried out to determine whether AMFs use nitrogen from the soil or from the host plant.

### Contribution of AMF on the Soil Carbon Cycle and C Sequestration

Arbuscular Mycorrhizae Fungi play an essential role in the global C cycle. AMF hyphae are involved in C translocation into the soil and provide a key link in the terrestrial C cycle (Finlay, 2008). Indeed, AMF is an efficient agent to improve carbon sequestration in a mechanism of translocation C away from the high respiratory activity around the root and into the soil aggregations (Zhu and Miller, 2003). It has been demonstrated that mycorrhizal roots create a sink demand for carbon. When the atmospheric CO_2_ increases, the allocation of C from the plants to AMF also increases and stimulates the growth of AMF (Drigo et al., 2010). This C demand is provided by the host plant from the C fixed through photosynthesis (Parihar et al., 2020). In addition, AMF extramatrical hyphae represent 20–80% soil microbial biomass which consists of 15% of soil organic C (Kabir et al., 1997; Leake et al., 2004). As discussed above, AMF plays also a critical in the formation and maintenance of soil aggregations through the production of Glomalin. This glomalin protects organic matter from microbial degradation, increases the hydrophobicity and stability of macro-aggregations, which control soil carbon loss and increase soil carbon stocks (C sequestration; Wilson et al., 2009; Rillig et al., 2010). More studies are needed to distinguish the role of AMF in the dynamics of soils carbon sequestration. This involves in particular determining the quantity of carbon fixed by the AMF because this lack of knowledge means that AMF cannot currently be included in the models of reducing the rate of atmospheric carbon. In addition, limited information is available on the regulation of carbon to nutrient exchange across the mycorrhizal interface.

### Contribution of AMF on Soil Trace Elements Transfer

Trace elements play roles in enzymatic activities involved in photosynthesis, oxidative respiration, protection against free radicals, or even lipid biosynthesis (Dominguez-Nuñez et al., 2016). It is known that AMF allows better absorption of low mobile trace elements in soils, such as potassium (K), calcium (Ca), magnesium (Mg), copper (Cu), zinc (Zn), iron (Fe), manganese (Mn), and cobalt (Co) (Garcia et al., 2016; Hashem et al., 2018). For instance, according to Krishna and Bagyaraj (1984), the level of Zn, Fe, and Mn is twice in mycorrhizal peanut plants compared to non-mycorrhizal plants. It has also been revealed that mycorrhizal inoculation improved Zn and Cu nutrition in soybeans and clover (Schoeneberger et al., 1989). However, when some of these elements are present in high quantities and therefore possess a toxic character, the mycorrhization can play a role in the protection of the plant, by strong retention of these elements (Liu et al., 2000). Besides trace elements, more research studies are needed on the role of AMF to synthesize or transport phyto-hormones (auxin, cytokinins, gibberellic acid, etc.) and antibiotics from plant to plant and from plant to soil microorganisms.

## Role of AMF on Improving Soil Biological Properties

Microorganisms are one of the most important soil components. These microorganisms interact between them and with their environment to contribute to the functioning of the soil and thus participate in the provision of ecosystem services necessary for our survival (plant production, purification of pollutants, etc.; Nielsen et al., 2011). Soil is therefore a continually active biological reactor where diverse biochemical reactions and essential ecological processes happen (solubilization of organic matter, the biogeochemical cycles of the elements, etc.; Gessner et al., 2010). The microbial activities in the soils contribute to its fertility through synergy between microorganisms, competition, and parasitism (Topalović and Vestergård, 2021). Within the soil, AMFs interact with a wide range of microorganisms to better improve soil fertility. It has been demonstrated that the secretions of AMF influence the composition and activity of microbial communities in the rhizosphere (Veresoglou and Rillig, 2012). The biological activities of AMF lead to the appearance of a positive, neutral, or negative relationship between AMFs and other soil microorganisms.

Many microbial components of the soil work synergistically with AMF, promoting the growth and protection of plants (Gryndler, 2000; Barea et al., 2002). The positive interactions involve the nutrient acquisition, biological control of root pathogens, improvement of plant tolerance to abiotic stresses, and soil fertility. AMF communities influence the physicochemical environment of the rhizosphere and control various soil microbial interactions (Alimi et al., 2021). Mycorrhization directly affects the quantity and quality of root exudates. These exudates influence the composition of the microflora of the rhizosphere (Baltrus, 2017). Table 1 shows some examples of interactions between AMFs and other microorganisms. However, these interactions depend on several factors, such as the amounts of phosphorus and nitrogen available (Larimer et al., 2014). This is confirmed by Wang et al. (2011) and Xu et al. (2018) who demonstrated a synergistic relationship between AMF and the bacterial (i.e., rhizobia) and fungal communities depends on N and P status in the soil. However, there is a lack of information and pending questions which need to be answered. How soil microorganisms may hamper or totally inhibit the activities and functioning of AMF? What is the role of AMF in the trophic chain? In another word, can AMFs subject to any kind of predation or parasitism from soil microorganisms? In addition, a study on AMF and free native nematode interactions and their impact on the development of cereal crops under water stress conditions are also needed.

**Table 1:** Examples of some interactions between AMF and soil microorganisms.

**Interactions AMF + microbes**	**Mechanisms**	**Effects**	**References**
AMF and *Pseudomonas fluorescens*	*Glomus intraradice* (AMF) stimulates the production of antibiotic (2,4-diacetylphloroglucinol) by *Pseudomonas fluorescens*.	The antibiotic protects the host plants against *Gaeumannomyces graminis*.	Ma et al., 2019
AMF and saprotrophic fungi	AMF increase the biomass of saprotrophic fungi.	Dissolution of soil organic matter into mineral matter.	Albertsen et al., 2006; Carteron et al., 2021
AMF and Gram-positive/negative bacteria	AMF has a deletion effect on certain Gram-positive and Gram-negative bacteria.	This interaction affects the production of bioactive metabolites and the decomposition of organic matter.	Welc et al., 2010
AMF and *Rhizobia*	AMF work in synergy with Rhizobia	Provide legumes woody and crop legumes (Faba bean) with essential soil nutrients	Chatarpaul et al., 1989; Xavier and Germida, 2002
AMF, *Rhizobia*, and phosphorus solubilizing microorganisms (PSM)	Tripartite relationship. Solubilize P by mineralization, low soil pH, chelation and production of phosphatase, organic acid and proton.	Improve host plant phosphorus uptake.	Afkhami and Stinchcombe, 2016; Kalayu, 2019; Nacoon et al., 2020
AMF and Mycorrhization Helper Bacteria (MHB)	MHB help: in the receptivity of the root to the AMF, in root-AMF recognition, in AMF growth, in the modification of the rhizospheric soil, and in the germination of AMF propagules.	Beneficial effect of bacteria on mycorrhizae. Improve soil fertility and nutrients uptake by the host plants.	Rigamonte et al., 2010
AMF and Plant Growth Promoting Rhizobacteria (PGPR)	AMF work in synergy with PGPR to stimulate Ammonia production, N fixation, solubilization of mineral phosphate, and other essential nutrients, production of plant hormones. Accumulate ascorbate peroxidase and glutathione peroxidase. Secrete organic acids responsible for dissolving phosphorus phytate mineralization and inorganic P solubilisation. PGPR found in AMF mycelia produce siderophore and indol acetic acid production.	Soil fertility and plant growth. Increase the diversity and abundance of soil parasite antagonists. Mitigate water deficit damage and improve water stress tolerance (i.e., *Cupressus arizonica*).	Linderman, 2000; Ahemad and Kibret, 2014; Vafadar et al., 2014; Battini et al., 2016; Moreira et al., 2020
AMF and *Frankia*	Synergistic interaction between AMF and *Frankia* (nitrogen-fixing actinobacteria).	Improve actinorhizal plants' height, the numbers and dry weight of root nodules, leaf area, shoot height, total biomass, and N and P leaf contents (i.e., *Alnus glutinosa*).	Oliveira et al., 2005
AMF and *Bacillus subtili*s	AMF stimulate the production of nitrate and nitrite reductase and nitrogenase activities and osmoprotectants such as glycine, betaine, and proline by *Bacillus subtili*s.	Increase shoot and root dry weight, nodule number, and leghemoglobin content.	Hashem et al., 2017

*AMF, Arbuscular Mycorrhizae Fungi*.

## Influence of Crop and Soil Management Practices on the AMF Functioning and Performing Various Soil Functions

Arbuscular Mycorrhizae Fungi improve soil health by improving its physical, chemical, and biological health. The previous sections have described its role in nutrient cycling and interaction with other soil microorganisms. However, agriculture practices significantly impact AMF communities and their performance, influencing various soil functions. This section will discuss some crop and soil management practices and their influence on AMF functioning and performing activities in the soil.

### Impact of Crop Management on AMF

To maintain and improve nutrient availability and soil health and minimize pest and disease incidence, farmers use management strategies, such as crop rotation and intercropping, to achieve sustainable agriculture production. These two practices influence the diversity, abundance, and functioning of AMF in the soil. For instance, crop rotation increases the number of AMF spore density and root colonization in maize, and it also increases wheat yield when the preceding crop is soybean or chickpea (Higo et al., 2013; Bakhshandeh et al., 2017). These positive effects are not the effects of leguminous nitrogen fixation but rather due to higher AMF activities in the soil (Bakhshandeh et al., 2017). However, crop rotation with non-mycorrhizal crops, such as the Brassica family, reduces the abundance of AMF in the soil and the symbiotic benefits conferred to the crops through the production of antimicrobial isothiocyanates (Valetti et al., 2016). Moreover, some plants are more mycorrhizal-dependent than others. For instance, maize crop is more mycotrope than soybean (Troeh and Loynachan, 2003; Wang et al., 2016), while wheat is considered a non-mycorrhizal or mycorrhizal plant species depending on the cultivar (Hetrick et al., 1993; Stefani et al., 2020). Wheat cultivars respond differently to AMF inoculation in terms of growth, root colonization, and carbon for nutrient exchange; depending on the age of a cultivar, old cultivars benefit more consistently from AMF than new cultivars that effectively exploit highly fertilized systems with less reliance on symbiosis (García de León et al., 2020). This mechanism is known as a mycorrhizal dependency, whereby AMF presence in the soil affects the growth response of plant species differentially (Kandhasamy et al., 2020). In addition, intercropping various plant species in similar conditions impacts the composition of the community and diversity of AMF and the plant diversity. Indeed, AMF is involved in transporting plant assimilates from the dominant species to plant species subordinated through the AMF mycelium network (Egerton-Warburton et al., 2007). Therefore, the presence of AMF impacts the type of vegetation, the relative abundance of plant species, and their diversity (Yadav et al., 2020). This mechanism operates through a change in the soil microorganisms and soil properties (soil structure; Liu L. et al., 2020). AMF influences soil microbiota through mycelium products and biophysical mechanisms, such as enmeshment and alignment. All these mechanisms occur in a complex interaction process that involves various factors in a cycling way. However, we need to carry more research studies at the field level to really understand the impact of plant biodiversity on AMF diversity and functioning.

### Impact of Soil Management Practice on AMF

Soil management practices have a significant impact on soil properties and microbial diversity. Mineral fertilizer, chemical pesticides, and herbicides applications are essential for crop nutrition, and they replenish the soil nutrients pool removed or harvested by crops, weed control, and pest management (Rana et al., 2019). However, high- and long-term inorganic fertilizer application reduces the plant's dependency on AMF, subsequently, mycorrhizal diversity, and abundance (Kour et al., 2020). This phenomenon can be explained by the fact that the symbiotic relationship between the plant and AMF is energetically costly for the plant; therefore, when the soil is rich in nutrients, plants allocate fewer carbohydrates to AMF, which ultimately affects the spore development and hyphae production (Tian et al., 2013). Soil phosphorus plays the most significant role in regulating plant mycorrhizal symbiosis (Kowalska et al., 2015). Thus, high P application negatively affects root colonization and AMF diversity (Cheng et al., 2013). On the other side, low fertilizer application optimizes the plant mycorrhizal symbiosis (Rana et al., 2020). For instance, Liu et al. (2016) found that P application in nutrient-deprived soil improves the mycorrhizal-mediated benefits to the plant. In addition, organic fertilizer application also has both positive and negative impacts on AMF diversity (Liu J. et al., 2020). According to Zhu et al. (2016), organic matter improves the AMF community composition in the rhizosphere of maize. In the same vein, inorganic pesticide and herbicide applications also have both positive and negative effects on AMF. These effects are mediated by the secretion of active substances up taken by plants via root or hyphal from the rhizosphere (Hage-Ahmed et al., 2019). For instance, azoxystrobin and glyphosate, respectively, fungicide and herbicide, inhibit the spore germination of some AMF species (Buysens et al., 2015). In addition to the chemical application, extensive tillage greatly influences the community composition of AMF by reducing mycelium extension, colonization rate, and diversity structure (Säle et al., 2015; Zhao et al., 2015). This is due to the effects of tillage on the soil's permeability, texture, and microbial food substrates, which ultimately affect the soil microbiota activity and their habitat type (Wang et al., 2020). Several studies demonstrated that conservation or zero tillage improves AMF diversity and abundance, resulting in better plant growth (Qin et al., 2017; Gu et al., 2020). However, a study carried out in a Mediterranean agroecosystem found that AMF spore density or extraradical mycelium density is not affected by conventional tillage practices (Curaqueo et al., 2011). These contradicting results indicate that the effects of different chemical applications and tillage practices on AMF still need further studies to understand better the effect of different soil management practices on soil AMF.

## Conclusion and Future Areas of Research

Arbuscular Mycorrhizal Fungi appear to be one of the most important soil organisms to take into account. AMFs are involved in plant mineral nutrition, water absorption, and protection against biotic and abiotic stresses in plants. Despite the fact that the importance of AMF in improving soil fertility is well-established, our understanding of the underlying mechanisms is still limited. There are a few studies that simultaneously investigated the effects of AMF on the physical, chemical, and biological properties of the soil. Therefore, the current review provides a holistic overview of the existing information regarding the role of AMF symbiotic relationships with crops in improving the physical, chemical, and biological properties of the soil. Regarding the impact of AMF on soil fertility, we highlighted several mechanisms, such as the production of glomalin, which is beneficial to the accumulation and circulation of soil carbon and enhances soil stability. In addition, the beneficial interaction between AMF with other soil microorganisms, such as PSB, which produce phosphatase and mineralize organic P, was highlighted. However, some of the functions involved in this symbiosis that determine the performance of AMF in the soil should be addressed in future studies.

- Future studies are required to characterize the soil P and N critical threshold below which AMF establishes symbiosis and above which AMFs are not active on a broader range of plant-AMF species combinations, soil types, and edaphic conditions.
- Furthermore, future research should investigate the regulation of N and its uptake from the soil during AMF symbiosis by using molecular tools, such as transcriptomic, gnomonic, and the development of fungal mutants.
- Further attention is needed on the role of glomalin in improving carbon sequestration efficiency from various climate and soil types to expedite its use in solving soil degradation problems that will be worsened by prevailing climate disturbances. Assessing the accumulation and lifespan of glomalin in soil fertility parameters under different climate, land use, and management conditions is of critical importance.
- It is well-established that the interactions between AMF and certain soil microorganisms are beneficial to soil fertility; however, the interaction between AMF and free native nematode and their impact on soil structure under drought stress calls further research.
- The role of AMF in the synthesis or transport of phytohormones, such as auxin, cytokinins, gibberellic acid, and antibiotics from plant to plant and from plant to soil microorganisms, is also poorly understood. Therefore, studies targeting the identification and characterization of such AMF function are paramount.
- Moreover, the role of AMF in soil basal respiration is an interesting field to investigate.
- Advances in our knowledge of the functions played by AMFs in the soil are partly hindered by the obligate biotrophic nature of these fungal microorganisms. Therefore, more field experiments on the impact of plant biodiversity on AMF diversity and functioning are necessary.
- Moreover, future studies on the effects of different soil management practices (i.e., tillage) and chemical applications on AMF functioning and performance in the soil are important.

All these research topics should be based on new approaches, such as recent methodological advances in physiology, molecular biotechnology, and agroecology integrated into both laboratory and field conditions. Such interventions are paramount to our ability to establish a new “green revolution” aligned to the requirements for achieving a sustainable development ingrained in agricultural production.

## Author Contributions

AFF contributed on inception of the paper, research, and writing. GN contributed on inception and reviews of the paper. JS contributed on inception and reviews. HFM contributed on inception and reviewed the work. SOA contributed on write up. AB contributed on write up and the revision of the manuscript. AN and KN reviewed the work. All authors contributed to the article and approved the submitted version.

## Funding

This work has been funded by Regional Academic Exchange for Enhanced Skills in Fragile Ecosystem Management in Africa (REFORM) grant number: 2017–2861.

## Conflict of Interest

The authors declare that the research was conducted in the absence of any commercial or financial relationships that could be construed as a potential conflict of interest.

## Publisher's Note

All claims expressed in this article are solely those of the authors and do not necessarily represent those of their affiliated organizations, or those of the publisher, the editors and the reviewers. Any product that may be evaluated in this article, or claim that may be made by its manufacturer, is not guaranteed or endorsed by the publisher.
